# Wheezing/asthmatic children: profile of children attended in the emergency department of the Hospital Geral de Nova Iguaçu (HGNI), Nova Iguaçu, RJ, Brazil

**DOI:** 10.1186/1939-4551-8-S1-A100

**Published:** 2015-04-08

**Authors:** Sylvia Gabriella Maia Araújo, Gisele Cardoso Nery, Daniella Mouta Dos Santos Silva, Raphael Coelho, Gustavo Henrique De Martin Ballini, Aniele Soares Moritz, Luiz Gustavo Bernardo De Oliveira, Sérgio Duarte Dortas

**Affiliations:** 1 Universidade Iguaçu, Brazil; 2 Hospital Universitário Clementino Fraga Filho Hucff-Ufrj, Brazil

## Background

Asthma is a chronic inflammatory disease of the lower airways, where episodes of wheezing are common causes of morbidity in the pediatric population.

We aim to describe the profile of pediatric patients treated at a regional reference emergency department (ED) with bronchospasm (CID: J45 and J42) from January 2008 to March 2014.

## Methods

A descriptive, cross-sectional study with retrospective data colletion from children attended at our ED. Analyzed parameters: age, race, sex, crisis type, medications, hospitalzation time, readmission and complications.

## Results

Data from 145 patients ranging from 7 days to 12 years, predominantly in patients less than 3 years (60%) were collected. Predominated (53.79%) were male. Regarding race, 72.4% brown, 20% white and 7.6% black. Mean hospitalization time was 4.5 days. Regarding the severity of the exacerbations, 60% of light /médium, 39% of severe attacks and 1% respiratory arrest imminent attacks. There were 21 cases of readmission (14.48%). The main associated complication has beenpneumonia (27.59%). The most common treatment (17.24%) was Hydrocortisone associated with nebulizedusing bronchodilators.

## Conclusions

Our study shows a prevalence of wheezing attacks on male children under 3 years old, as described by other authors. We consider indispensable to educateparents and caregivers about the importance of prevention of Asthma. Thus avoiding readmission, as seen in ourpopulation (≈ 30%).

